# Microsporidial Keratoconjunctivitis Caused by *Vittaforma corneae*, Sea of Galilee, Israel, 2022–2024

**DOI:** 10.3201/eid3108.241941

**Published:** 2025-08

**Authors:** Asaf Friehmann, Irit Lubitz, Fidaa El Zhalka, Sharon Amit

**Affiliations:** Meir Medical Center, Kfar Sava, Israel (A. Friehmann, F. El Zhalka); Tel-Aviv University, Tel-Aviv, Israel (A. Friehmann, S. Amit); Sheba Medical Center, Ramat-Gan, Israel (I. Lubitz, S. Amit).

**Keywords:** *Vittaforma corneae*, microsporidia, fungi, keratoconjunctivitis, waterborne outbreak, Israel

## Abstract

We describe a multiannual outbreak of keratoconjunctivitis caused by the microsporidium *Vittaforma corneae* in the Sea of Galilee, Israel. Patients had multifocal punctate corneal infiltrates and reduced visual acuity, confirmed by locally-developed pathogen-specific real-time PCR. Topical chlorhexidine, rather than traditional antimicrobial drugs, proved an effective and safe primary treatment.

Microsporidial keratitis, caused by spore-forming unicellular parasites now classified as fungi, has previously been recognized as a severe yet uncommon ocular infection, often associated with outbreaks worldwide (*1*,*2*). *Vittaforma corneae* (previously known as *Nosema corneum)* was first identified in a child from Sri Lanka in 1973 (*3*) and has emerged as a keratoconjunctivitis pathogen, especially in water-related outbreaks. *V. corneae* is characterized by small spores (3–4-μm long and 1–1.5μm wide), has unique ultrastructural features, and exhibits a specific affinity for ocular tissues.

The natural reservoir of *V. corneae* remains unknown, yet it has been detected in various mammals and invertebrates, suggesting a broad host range. Of note, humans are not considered natural hosts of *V. corneae*. Environmental sources, particularly aquatic environments, are believed to play a crucial role in *V. corneae* transmission and persistence. Studies have identified *V. corneae* spores in both fresh and marine water samples, indicating its ability to survive in diverse aquatic settings (*1*,*2*).

In this article, we describe a multiannual outbreak of *V. corneae* keratoconjunctivitis associated with exposure to the Sea of Galilee in northern Israel. This outbreak is noteworthy for its prolonged duration and specific geographic association, which has not been previously reported for *V. corneae* infections. Our PCR-confirmed cases of *V. corneae* keratoconjunctivitis represent dozens of instances nationwide. This study was approved by the Meir Medical Center Internal Review Board (approval no. 0001-24-MMC).

## The Study

During 2022–2024, we detected 12 PCR-confirmed patients, 6–51 years of age (median age 15.3 years; mean ± SD age 22.29 ± 19.76 years); 5 were female and 7 were male. All reported recent exposure to the Sea of Galilee before symptom onset, with a median duration of 14 days (range 10–18 days) and an average of 13.75 days (SD ± 2.33 days) between exposure and symptoms. Similar to other nationwide reported cases, no other epidemiologic links were identified (Appendix).

Patients reported eye symptoms of redness, watering, irritation, and a foreign-body sensation. Visual acuity when seeking care averaged 0.60 ± 0.34 decimal (normal vision >1.0 decimal). Slit-lamp examination revealed coarse, multifocal, punctate epithelial lesions (<1 mm) on the cornea, often accompanied by nonpurulent conjunctivitis with a mixed follicular-papillary reaction (Figure). Those findings were uniform across the patient cohort and consistent with previous descriptions of *V. corneae* infections.

**Figure:**
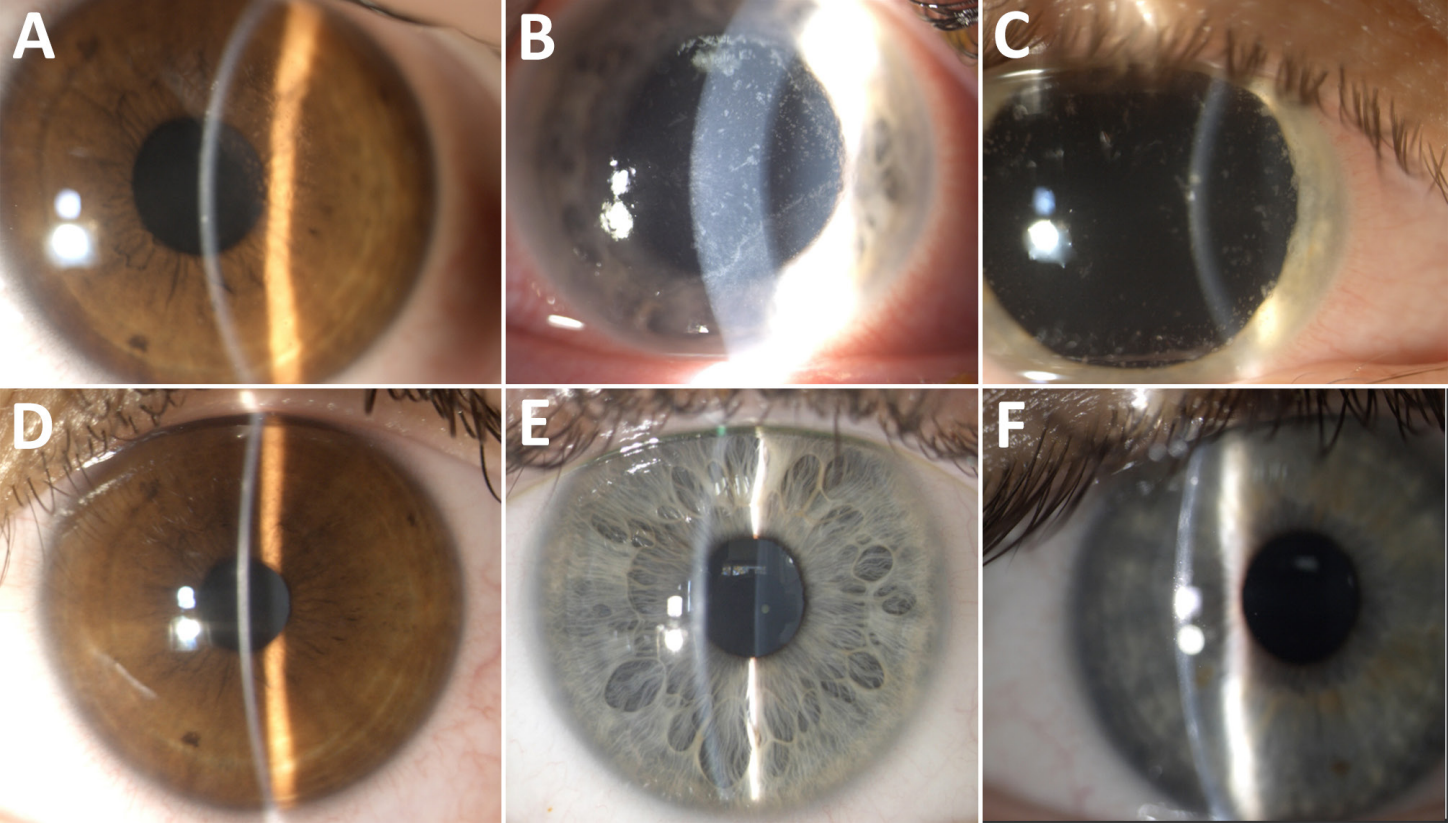
Slit-lamp photos of 3 patients before and after treatment of microsporidial keratoconjunctivitis caused by *Vittaforma corneae*, Sea of Galilee, Israel, 2022–2024. A) Patient 1 before treatment, visual acuity 0.8 decimal. B) Patient 2 before treatment, visual acuity 0.3 decimal. C) Patient 3 before treatment, visual acuity 0.3 decimal. D) Patient 1 after treatment, visual acuity 1 decimal. E) Patient 2 after treatment, visual acuity 1 decimal. F) Patient 3 after treatment, visual acuity 1 decimal. Clinical manifestations in all 3 patients included corneal epithelial microsporidial infiltrates and conjunctival irritation. After treatment with 0.02% topical chlorhexidine, the infiltrates resolved without scarring or other complications.

During the early months of the outbreak, the causative organism was unidentified. The ophthalmologic findings matched those of previously reported microsporidial infections and were supported by microscopy of corneal scraping specimens. We found numerous oval spores 3–5µm long and 1–2µm wide (*4*; https://www.cdc.gov/dpdx/microsporidiosis/index.html) by using fluorescent calcofluor staining. We used a pan-microsporidial PCR targeting the small subunit rRNA of most microsporidia (ss18f, 5′-caccaggttgattctgcc-3′; ss1492r, 5′-ggttaccttgttacgactt-3′) (*5*), but it failed to identify any specific pathogen, likely because of limited corneal scraping material and other technical limitations, such as primer mismatches with the target species. Next, we performed a shotgun metagenomic sequencing on pooled corneal scrapings by using a Nextera XT library and Illumina MiSeq platform (Illumina, https://www.illumina.com), yielding >7 million reads. Despite a predominance of host-derived sequences, species-specific reads mapping to the *V. corneae* small subunit rRNA gene verified the presence of *V. corneae*. To confirm this result, we developed a SYBR Green–based real-time PCR assay for *V. corneae*, following the methodology outlined previously (*6*). The assay used the primer sequences corn-F 5′-ctaccaagacagtgacggttga-3′ and corn-R 5′-ggcatcttttactgctggaact-3′. We conducted Sanger sequencing on the amplicons, yielding 100% coverage and >95% identity with *V. corneae*. 

Our key finding was the efficacy of topical chlorhexidine as a first-line treatment after corneal debridement, which was therapeutic and diagnostic. All patients were treated with 0.02% chlorhexidine 2–3 times daily, demonstrating excellent tolerability and outcomes. This regimen marks a shift from the combination therapies (sometimes including systemic drugs) typically used for microsporidial keratitis (*7*,*8*).

In contrast, only 3 patients in our study received either topical moxifloxacin or topical voriconazole (2–3 times daily), and 3 were treated with short courses of adjunctive topical steroids during follow-up (Appendix Table 1). Of note, no patients required hospitalization or additional interventions beyond topical therapy. Chlorhexidine was well tolerated, and no major corneal scarring or reported ocular discomfort related to its use was reported. Those findings support the potential of a simplified, topical-only approach to treatment.

Visual acuity improved in most patients, and the mean at the last follow-up (average 6.1 ± 4.2 months) reached 0.87 ± 0.21 decimal visual acuity. Although not statistically significant (p = 0.1) because of the small sample size, the improvement in visual acuity does indicate that topical chlorhexidine is effective in preserving visual function and preventing disease progression.

## Conclusions

In this article, we present a large outbreak of PCR-confirmed *V*. *corneae* keratoconjunctivitis associated with a single freshwater body, the Sea of Galilee, which has not previously been linked to a microsporidial outbreak. The multiannual nature of this outbreak, spanning >3 consecutive years, suggests the presence of a persistent environmental reservoir of *V. corneae* in this ecosystem, potentially influenced by unique ecologic conditions or anthropogenic factors. This outbreak is of public health interest given the widespread recreational use of the Sea of Galilee, which is the only major freshwater lake in Israel.

Our findings underscore the effectiveness and safety of topical chlorhexidine as a treatment for *V. corneae* keratoconjunctivitis. This simple, cost-effective regimen achieved favorable outcomes without the need for complex multidrug therapies or hospitalization. Although chlorhexidine avoids unnecessary exposure to systemic antimicrobial drugs and offers broad-spectrum coverage, this regimen may be insufficient in more complicated cases, such as contact lens–related infections involving *Pseudomonas* spp., other more complex pathogens, or immunocompromised hosts. Corneal scarring did not develop in any of our patients, suggesting chlorhexidine can preserve corneal integrity.

Early and accurate diagnosis was essential for guiding appropriate treatment. Because of the rarity of microsporidial infections in Israel, a species-specific real-time PCR enabled rapid, reliable detection of *V. corneae* from limited ocular samples. PCR proved especially useful in settings where microsporidial keratitis was not routinely suspected, enabling timely therapy.

The first limitation of this study is that, apart from 1 patient with untreated stable sarcoidosis, all patients were immunocompetent, limiting applicability to immunocompromised populations who may require more intensive treatment and prolonged follow-up and whose keratitis could be associated with systemic infection (*9*). Finally, the restriction of the outbreak to a single, ecologically unique body of water, the Sea of Galilee, limits the broader generalizability of these findings.

Further research is needed to clarify the ecologic and microbiological factors contributing to the persistence of *V. corneae* in the Sea of Galilee and to assess the potential for similar outbreaks elsewhere. This event also emphasizes the need for public health measures, including environmental monitoring and preventive recommendations, highlighting an emerging pattern in waterborne microsporidial infections, and the need for increased awareness among clinicians and microbiologists. PCR was essential for rapid and accurate pathogen identification, and the success of chlorhexidine 0.02% as a primary therapy offers a promising, simplified approach for managing such infections.

Appendix Additional information for microsporidial keratoconjunctivitis caused by *Vittaforma corneae*, Sea of Galilee, Israel, 2022–2024.
