# Supplementary material for: Microsporidial Keratoconjunctivitis Caused by Vittaforma corneae, Sea of Galilee, Israel, 2022–2024
Source: Emerg Infect Dis. 2025 Aug;31(8):1645–7. doi: 10.3201/eid3108.241941 (PMC12309766; doi:10.3201/eid3108.241941)
Supplement: Appendix — Additional information for microsporidial keratoconjunctivitis caused by Vittaforma corneae, Sea of Galilee, Israel, 2022–2024. [file 24-1941-Techapp-s1.pdf]

Article DOI: https://doi.org/10.3201/eid3108.241941

EID cannot ensure accessibility for supplementary materials supplied by authors. Readers who have difficulty accessing supplementary content should contact the authors for assistance.

Microsporidial Keratoconjunctivitis Caused by *Vittaforma corneae*, Sea of Galilee, Israel, 2022–2024

Appendix

Appendix Table. Demographic and clinical characteristics of patients in the cohort

Patient	Sex	Age, years	Time from exposure to symptoms, days	Visual acuity on admission (decimal)	Visual acuity at last visit (decimal)	Immune status	Primary Topical Agent	Adjunct Topical Therapy	Follow-up (weeks)
1	Male	51	14	0.2	0.5	Immunocompetent	Chlorhexidine	NA	48*
2	Male	18.3	10.5	0.3	0.66	Immunocompetent	Chlorhexidine	NA	1
3	Female	12.2	18	0.3	1	Immunocompetent	Chlorhexidine	Steroids	2
4	Male	18	13	1	1	Immunocompetent	Chlorhexidine	Steroids	6
5	Female	12.6	14	0.8	1	Immunocompetent	Chlorhexidine	NA	16
6	Male	9.1	16	0.8	NA†	Immunocompetent	Chlorhexidine	NA	NA†
7	Male	71	12.5	0.4	0.5	Untreated, stable sarcoidosis	Chlorhexidine	Moxifloxacin	3
8	Female	15.1	10	1	1	Immunocompetent	Chlorhexidine	NA	12
9	Male	15.5	16	1	1	Immunocompetent	Chlorhexidine	Voriconazole + Steroids	12
10	Female	10	14	1	1	Immunocompetent	Chlorhexidine	NA	4
11	Male	6	12	1	1	Immunocompetent	Chlorhexidine	NA	4
12	Female	36	15	0.3	1	Immunocompetent	Chlorhexidine	Moxifloxacin	4

All patients presented with varying degrees of typical symptoms, including redness, tearing, irritation, and foreign body sensation.
*The prolonged follow-up of Patient 1 in the ophthalmology clinic was due to unrelated retinal issues.
†Lost to follow-up.
